# A Practical Compendium of the Materia Medica; with Numerous Formulæ Adapted for the Treatment of the Diseases of Infancy and Childhood

**Published:** 1838-10

**Authors:** 


Art. VII.—*A Practical Compendium of the Materia Medica; with numerous Formulæ adapted for the Treatment of the Diseases of Infancy and Childhood.* By Alexander Ure, m.d., Member of the Royal College of Surgeons.—*London,* 1838. 12mo. pp. 221.

In compiling this little work, the author has followed the plan of Professor Fränkel, in his "Practical Therapeutics for the Diseases of Childhood;" and has produced a book which will be useful both to the student and practitioner. The various articles of the materia medica are here treated of principally in their application to the complaints of children; and numerous formulæ for their administration are given, derived from various sources. In a Preliminary Dissertation, general therapeutical agents are fully discussed; as bloodletting (local and general), bathing, emetics, enemata; and the modifications necessary in the employment of them, in consequence of the tender age of the patient, are well explained. Dr. Ure has enriched his work with many formulæ derived from German authors. The more important articles of the materia medica, and those more generally in use in the diseases of childhood, are very fully treated of; and all the cautions necessary in the administration of calomel, opium, and the poisonous remedies are mentioned, so as to render the work at once a safe and useful manual in the class of diseases to which it is especially devoted. We strongly recommend it to those commencing general practice, to whom the diseases of infants are often a stumbling-block.

